# Internal tsunami waves transport sediment released by underwater landslides

**DOI:** 10.1038/s41598-019-47080-0

**Published:** 2019-07-24

**Authors:** Noel Brizuela, Anatoliy Filonov, Matthew H. Alford

**Affiliations:** 1 Scripps Institution of Oceanography, University of California San Diego, La Jolla, 92093 USA; 2 Department of Physics, Universidad de Guadalajara, Guadalajara, 44430 Mexico

**Keywords:** Sedimentology, Fluid dynamics, Natural hazards, Geomorphology, Physical oceanography

## Abstract

Accelerated by gravity, submarine landslides transfer energy to the marine environment, most notably leading to catastrophic tsunamis. While tsunamis are thought to use less than 15% of the total energy released by landslides, little is known about subsurface processes comprising the rest of their energy budgets. Here, we analyze the first set of observations depicting a lake’s interior response to underwater landslides and find that sediment transport is modulated by baroclinic waves that propagate along vertical gradients in temperature and sediment concentration. When traveling along a shallow thermocline, these waves can reach past topographic features that bound turbidity currents and thus expand the influence area of underwater landslides. With order of magnitude calculations, we estimate that observed thermocline internal waves received roughly 0.7% of available landslide energy and infer their contribution to homogenize the lake’s thermodynamical properties by means of turbulent mixing. Lastly, we show that landslides in our data set modified the lake’s intrinsic dynamical modes and thus had a permanent impact on its circulation. This suggests that measurements of subsurface wave propagation are sufficient to diagnose bathymetric transformations. Our experiment constitutes the first direct observation of both internal tsunami waves and turbidity current reflection. Moreover, it demonstrates that background density stratification has a significant effect on the transport of sediment after submarine landslides and provides a valuable reference for numerical models that simulate submarine mass failures.

## Introduction

Tsunamis are reported at an average rate of one per year when surface waves generated by impulsive geophysical events flood coastal regions^1^. Although tsunamis are most commonly caused by earthquakes, underwater landslides have gained prominence as a generation mechanism since the discovery of their role in modern-day catastrophes such as the Papua New Guinea and Tohoku tsunamis of 1998 and 2011^1–4^. More recently, early observations have attributed the tragic Sulawesi^5^ and Sunda Strait tsunamis of 2018 to coastal sediment failures. Despite large scientific interest, the lack of in situ observations and the temporal sparseness of records have delayed progress in our mechanistic and probabilistic understanding of underwater landslides as a natural hazard^6^. The generation of surface waves by underwater landslides is thought to account for less than 15% of total landslide energy^7^, but represents the largest threat for human populations and is thus the priority of numerous idealized experiments and numerical simulations. Meanwhile, subsurface processes that control the resuspension, transport and deposition of sediment in these events remain uncertain, and the impacts of a background density stratification have yet to be established^8–10^.

Internal Gravity Waves (IGWs), which arise when density surfaces in a stratified fluid are perturbed by external forces, are an overlooked consequence of sudden seafloor deformation^11^. Commonly generated by tides and wind, these motions are known to redistribute energy over global scales^12,13^ and sediment along the continental margin^14^, ultimately impacting climate^15^ and possibly shaping continental slopes^16^. IGWs are often measured with moored thermistors, and appear in time series as periodic temperature fluctuations whose magnitude is used to infer vertical displacements in the water column. Until now, such observations of internal tsunami waves (IGWs generated by impulsive geophysical events) are lacking, as tsunamigenic processes are largely unpredictable and can easily wreck oceanographic instruments. Although perceived as lacking the destructive potential of their surface counterparts, internal tsunamis may play a major role in the transport of mass following submarine landslides and hence pose an imminent threat to deep sea ecosystems^17^.

**Figure 1:**
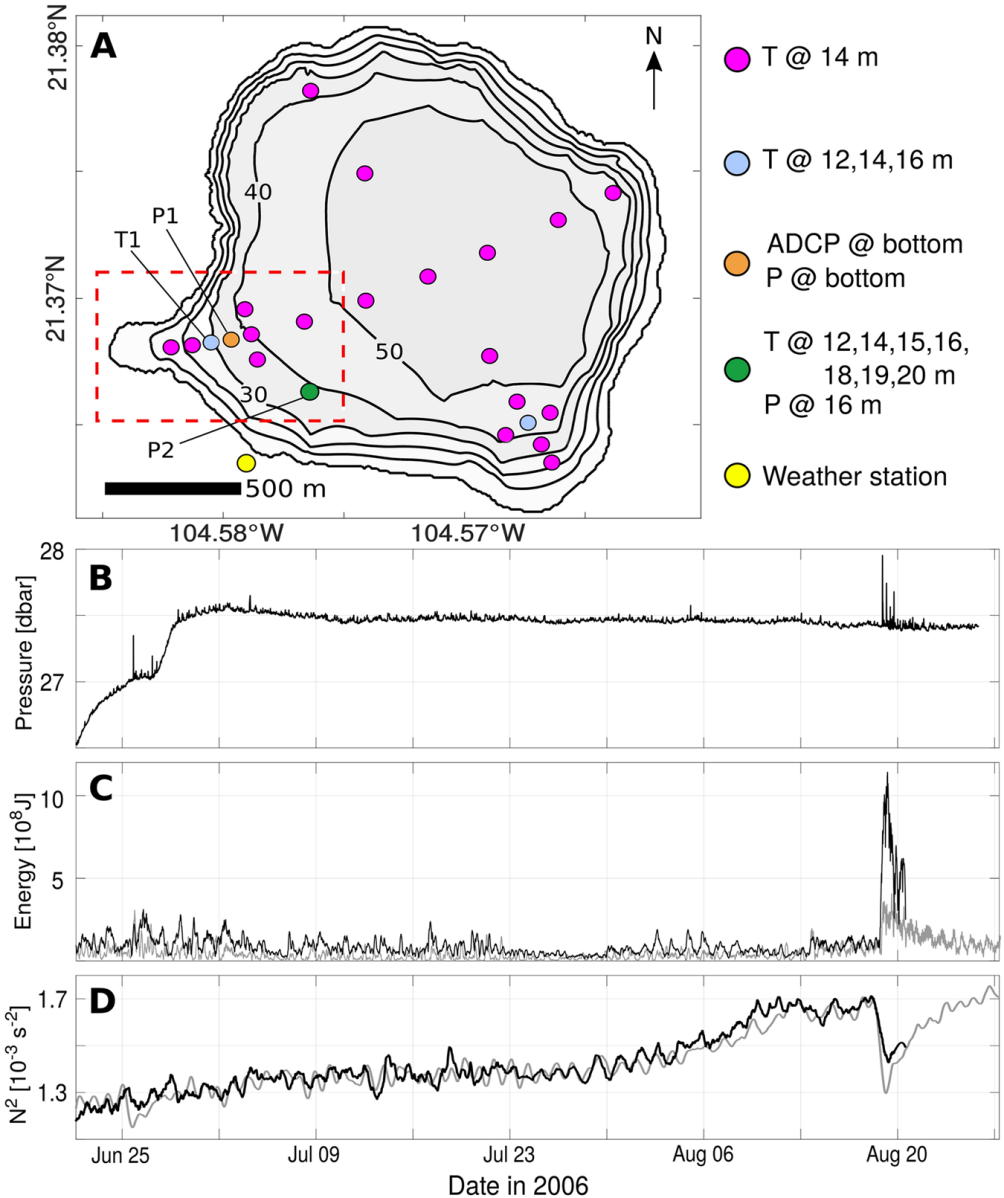
Experiment overview: Panel (A) shows the locations of ADCP, thermistors (T), pressure sensors (P) and a weather station deployed over the summer of 2006. The red, dashed rectangle indicates the location of Agua Caliente Bay (Fig. 2). Slow trends and rapid spikes in the time series of bottom pressure at P1 (**B**) reveal the occurrence of landslides in late June and mid August. Linear (gray) and nonlinear (black) estimates of energy stored in thermocline oscillations ((**C**) see Methods) show that internal motions following the landslide of August 18 were many times more energetic than the lake’s wind-driven circulation. Panel (D) shows the squared buoyancy frequency ${N}^{2}=-\,\frac{g}{\rho }\frac{\partial \rho }{\partial z}$ as measured by our P2 chain (black) and as predicted by a linear model ${N}_{P2}^{2}=1.6{N}_{T1}^{2}$ that uses measurements of *N*^2^ at T1 (gray). Both time series have been low-pass filtered with a 24-hour cutoff.

**Figure 2:**
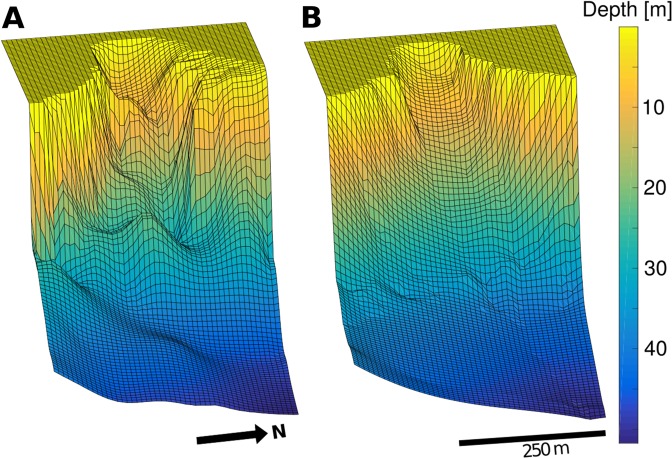
Santa María del Oro before and after: Single-beam acoustic measurements of water depth made in 2000^18^ (**A**) and a composite of data obtained between 2009 and 2016 (**B**) have been interpolated to produce these surfaces. Despite the coarse spatial resolution of our data (see Supplementary Fig. 1), differences between surveys and scars near 30 m depth in Panel (B) suggest the occurrence of underwater landslides in Agua Caliente Bay (red, dashed rectangle in Fig. 1A).

The volcanic lake Santa María del Oro (SMO, Western Mexico; Fig. 1A) is strongly stratified during summer and forced year-round by a diurnal wind cycle that induces seiches on its surface^18^. At the thermocline level, atmospheric forcing prompts modal IGWs that circulate the lake at frequencies dictated by its geometry and thermal stratification. Consequently, the lake’s IGW field evolves with the seasons and would also, according to theory, be altered by changes in topography. SMO underwent severe geomorphic transformations between 2000 and 2016, including a series of landslides that reshaped its Agua Caliente Bay (Fig. 2). Scars at the bottom of the 6% slope that now occupies the bay evidence these failures, but cannot reveal their timing. However, 32 thermistors, 2 pressure sensors and one Acoustic Doppler Current Profiler (ADCP) deployed over the summer of 2006 measured the basin’s immediate and long-term response to a series of landslides in this area (Fig. 1). In this article, we analyze the resulting data and focus on the generation of IGWs by underwater landslides, the periodic behavior of a subsequent turbidity current, and the long-term dynamical effects of basin transformations.

## Results

**Figure 3:**
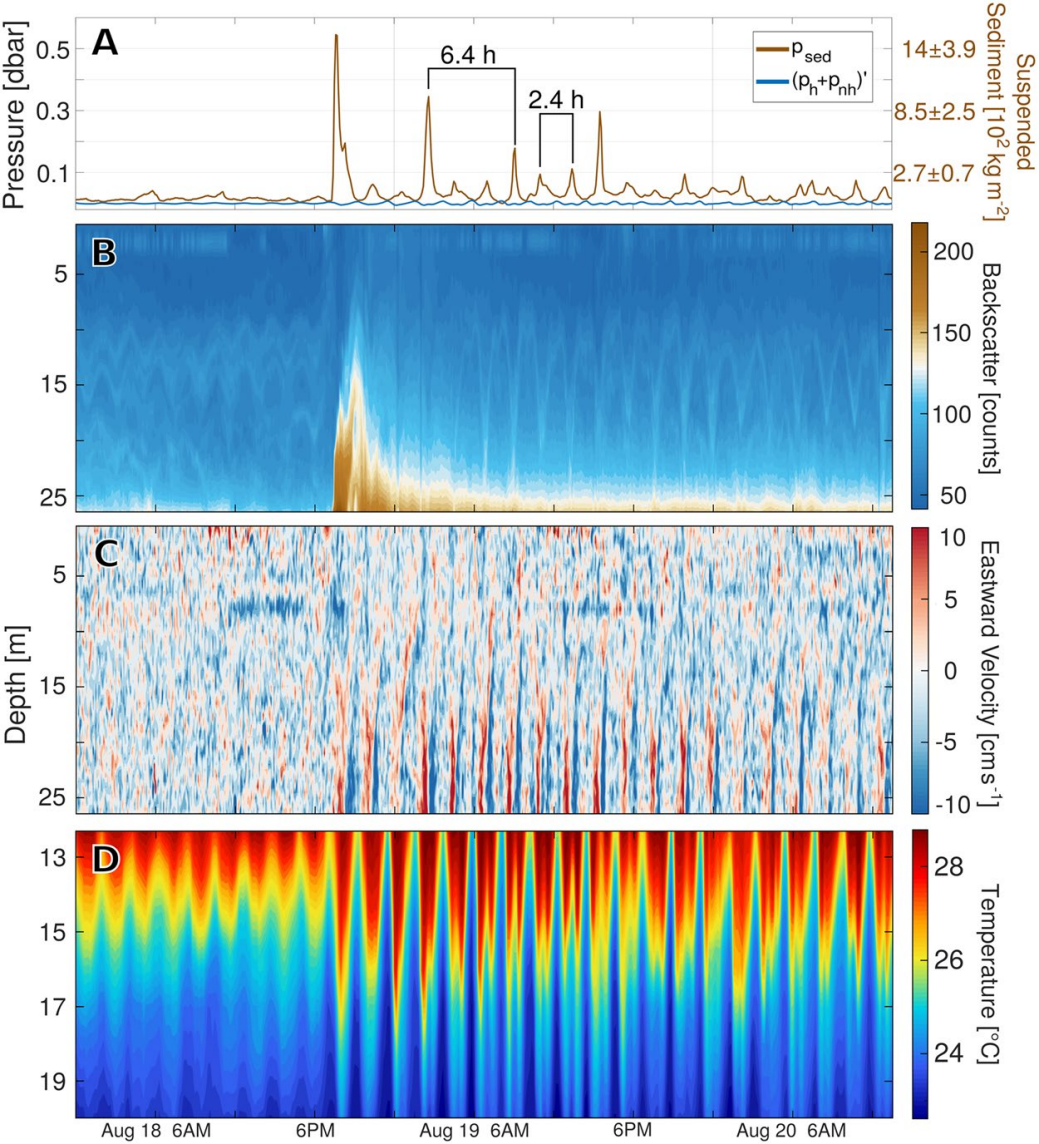
Hydrodynamics of the August 18 landslide: Panel (A) shows fluctuations in bottom pressure attributed to resuspended sediment *p*_*sed*_ and hydrodynamic processes $({p}_{h}+{p}_{nh})^{\prime}$ (left axis). The equivalence of *p*_*sed*_ to vertically-integrated sediment concentrations was calculated using ${\rho }_{sed}=1700\mp 300\,{{\rm{kgm}}}^{-3}$ (Eq. 2, see Methods) and is shown on the right axis. ADCP values of acoustic backscatter in panel (B) peak with *p*_*sed*_, confirming enhanced sediment concentrations. Profiles of velocity at P1 (**C**) and temperature at P2 (**D**) demonstrate the lake’s interior response to the landslide and subsequent forcing by turbid internal seiches.

Records of bottom pressure at P1 that have been adjusted for changes in the lake’s water level (Fig. 1B, see Methods) show a positive trend over the first 11 days of our experiment, when the lakebed deepened locally by as much as 1.1 m. Throughout this period, five distinct regimes of sediment motion are characterized by local subsidence rates that range between 0 and 0.32 m day^−1^ (Supp. Fig. 2, Supp. Table 1). Likewise, rapid spikes in bottom pressure are evidence of turbidity currents and underwater landslides, as large concentrations of suspended sediment exert an additional load on our sensor when they transit through P1. We used ADCP and thermistor data taken at P1 and P2 to estimate the contributions made to bottom pressure by variations in the lake’s thermal structure *p*_*h*_ and non-hydrostatic effects *p*_*nh*_ (see Methods) and subtracted those from our measurements *p*_*m*_. The remaining signal ${p}_{sed}={p}_{m}-({p}_{h}+{p}_{nh})$ can be used to infer the vertically-integrated concentration of sediment above P1 (Fig. 3A, see Methods). Given that suspended matter increases the acoustic reflectivity of water, ADCP measurements of acoustic backscatter further reveal the vertical extent of sediment clouds (Fig. 3B), enabling us to estimate their mean density.

**Figure 4:**
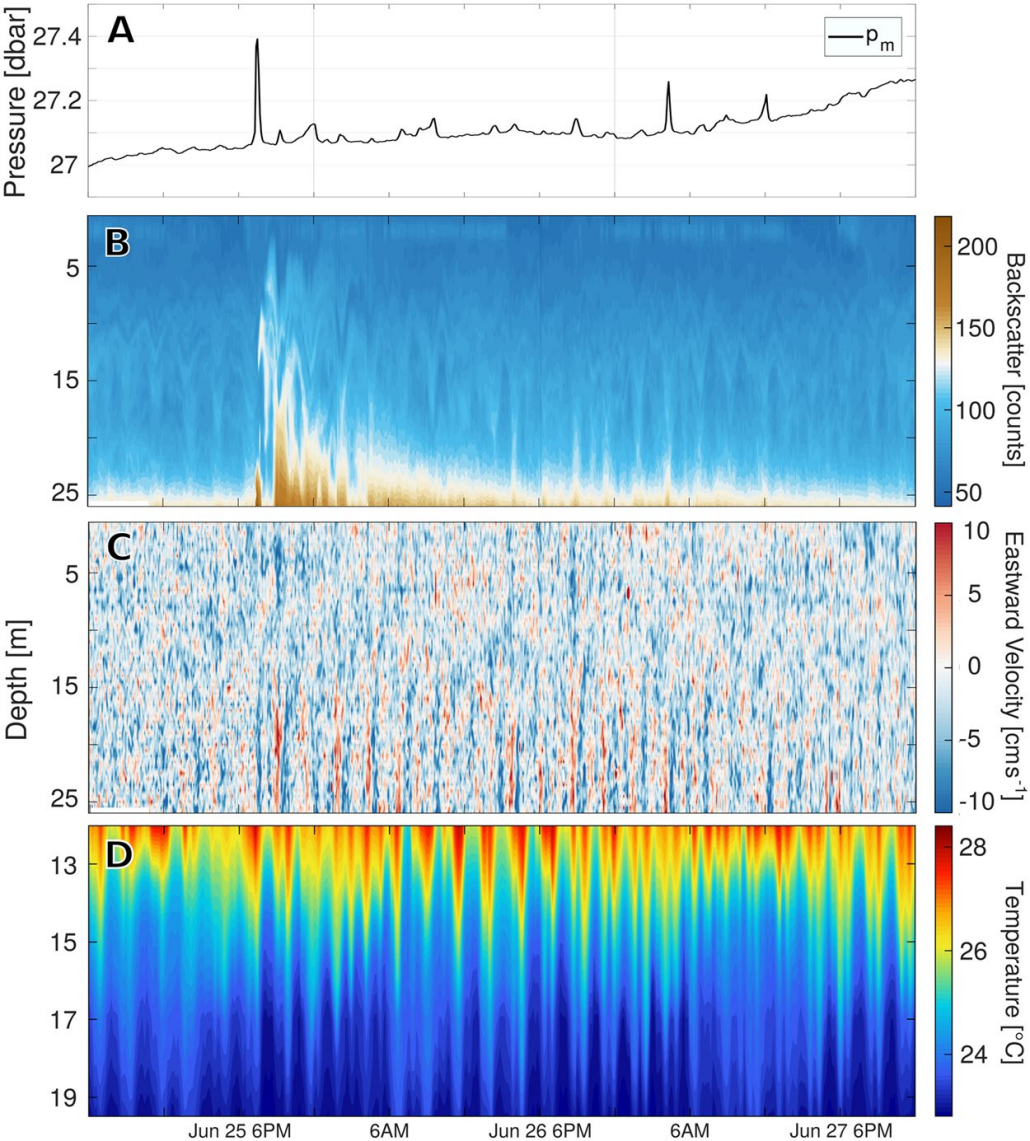
Hydrodynamics of the June 25 landslide: Panel (A) shows measurements of bottom pressure at P1 that have been adjusted for changes in the water level. Given vertical displacements of our pressure sensor throughout this period, measurements could not be decomposed into components *p*_*sed*_, *p*_*h*_, *p*_*nh*_. Anomalous values of ADCP acoustic backscatter (**B**) are associated with large disturbances in bottom pressure. These measurements reveal two separate branches of sediment transport, one following the lake’s bottom and another propagating along the thermocline. Profiles of eastward velocity at P1 (**C**) and temperature at P2 (**D**) show the lake’s baroclinic response to this landslide.

**Figure 5:**
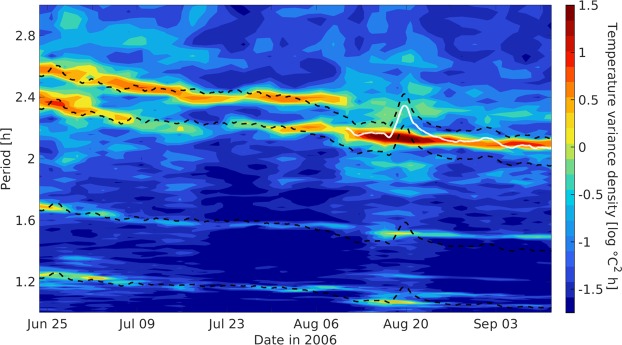
Spectrogram of thermistor data. Color shading shows the dominant periods of thermocline IGWs throughout our experiment. At each time, we use 8 days of temperature data to compute power density spectra for each sensor deployed at 14 m depth and plot the spectrum averaged between all instruments. Dashed lines show the theoretical periods $T=\frac{L}{N}\sqrt{{(\frac{2\pi }{L})}^{2}+{(\frac{n\pi }{h})}^{2}}$ of mode 1 (n = 1) IGWs with wavelengths L = 4150, 3800, 2720 and 2000 m (top to bottom) in a basin with constant depth h = 40 m. Before the landslide of August 18, the temporal variability of measured *N*^2^ (Fig. 1D) sufficed to produce a good agreement between theory and observations. After the landslide, however, our fits fail and most IGW energy shifts towards an oscillatory mode best described by L = 4020 m (white line).

While measurements of bottom pressure and acoustic backscatter demonstrate the occurrence of underwater landslides on June 25 (Fig. 4) and August 18 (Fig. 3), vertical profiles of temperature and flow velocity detail the hydrodynamic processes tied to them. During the August 18 landslide, a turbid front with an approximate mean sediment concentration of 190 ± 50 kg m^−3^ (Fig. 3A,B) accelerated downslope in Agua Caliente Bay, forcing near-bottom flows and perturbing the lake’s density stratification. As a result, high amplitude IGWs propagated through the lake’s thermocline with periods ranging between 1.1 and 2.4 h (Figs 3D and 5). Peaks that appear at 2.4 h intervals in the time series of *p*_*sed*_ reveal that thermocline IGWs conveyed sediment at concentrations near 260 ± 70 kg m^−2^ even 48 h after their generation (Fig. 3A). Estimates of available potential energy (APE) made using measured temperature (see Methods) indicate that the energy of thermocline oscillations peaked between 4 and 11 × 10^8^ J roughly 10 h after the landslide (Fig. 1C). The spread in our calculations is an effect of assumptions made about the lake’s vertical thermal structure and suggests that as much as 60% of the total observed APE was nonlinear. However, these values necessarily underestimate the true APE, since they don’t account for potential energy stored in sediment clouds, which are coupled to thermocline oscillations and can span the full water column (Figs 3B and 4B).

Large (*p*_*sed*_ > 0.15 dbar), periodic anomalies in the time series of sediment load (Fig. 3A) show that sediment suspended by the landslide of August 18 persistently returned to P1 in the form of near-bottom, high-density flows. As these basal motions reached Agua Caliente Bay and their pressure signature peaked, return flows intensified above them and ultimately generated a secondary phase of high-frequency thermocline IGWs roughly 7 h after the landslide (Fig. 3C,D). The recirculation of sediment at constant intervals of 6.4 h unequivocally points to the reflection of turbidity currents by the lake’s steep walls. Namely, waves of turbidity traveled back and forth between Agua Caliente Bay and the opposite end of the lake, where topography acted to reverse their propagation. This basin-wide, turbid oscillation remained active for a minimum of four cycles (25.6 h), implying that turbid fronts endured 8 reflections or more. Such temporal persistence requires sediment to propagate in the form of internal seiches along a vertical gradient in the concentration of suspended matter, as turbid solitons cannot endure multiple reflections^19^.

The organization of suspended sediment into a thin basal layer is predicted by theory^9^ and further evidenced by the confinement of enhanced acoustic backscatter at the bottom of the water column (Figs 3B and 4B). Unfortunately, our ADCP cannot sample within 1 m of the lakebed, and thus does not capture the core of these basal flows. Coupling between this near-bottom turbid layer and the preexisting two-layer temperature structure results in a three-layer system that allows the modulation of *p*_*sed*_ by thermocline IGWs (Fig. 3A). Additionally, this interaction allows turbidity currents to continuously power thermocline IGWs, whose energy peaked many hours after the landslide (Fig. 1C). Given its baroclinic nature, this process cannot be reproduced by numerical models that study turbidity currents interacting with constant density flows.

## Discussion

Recently, Sawyer and collaborators^17^ proposed that internal tsunami waves triggered by submarine landslides can transport salt out of brine pools and mix it with the environment above. Vertical mixing powered by the landslide of August 18 can be estimated using our calculations of internal tsunami wave energy as observed by the P2 thermistor chain (Fig. 1C). Throughout a 24-hour period following the slide, this estimate of thermocline energy decreased at an average rate of 3.4 ± 0.3 × 10^8^ J day^−1^, consistent with a basin-averaged dissipation rate of $\varepsilon =3\pm 0.3\times {10}^{-8}\,{\rm{W}}\,{{\rm{kg}}}^{-1}$ (the total lake volume was estimated to be 1.3 × 10^8^ m^3^). Similar to typical continental shelf values and on the high side of open-ocean measurements^20,21^, this rate of turbulent dissipation can help explain a sudden decrease of 17% in the lake’s density stratification from a pre-landslide condition of *N*^2^ = 1.7 × 10^−3^ s^−2^ to 1.4 × 10^−3^ s^−2^ less than 24 hours after the slide (Fig. 1D). Such vigorous mixing could not be sustained for long, as the lake restratified within 4 to 5 days. Overall, these observations support the view that landslide-generated IGWs could transport brine out of abyssal basins and produce enough turbulence to mix hypersaline waters into the deep ocean.

Past studies have proposed interfacial mixing between turbidity currents and the overlying flow as a source of anomalous materials in landslide deposits^22^. Our observations show that turbid internal seiches can be sustained for multiple cycles and thus drive a mode of sediment deposition that is characterized by alternating bidirectional flows. This mechanism is consistent with the stratigraphy of onshore surge deposits linked to the Chicxulub meteoritic impact^23^, whose truncated flame structures reveal vertical mixing generated via Kelvin-Helmholtz instabilities in the highly-stratified surge flow. Moreover, the existence of large, coherent sediment wave fields near oceanic slopes^24^ suggests that the horizontal advection of mass by turbid internal waves can significantly alter the geologic record over regional scales. Evidence presented in Fig. 3 exemplifies interaction between turbid gravity currents and the overlying density stratification, suggesting that numerical models must consider the background ocean as density-stratified in order to accurately reproduce all these sedimentary processes.

Density stratification is weaker over most of the ocean than it is in SMO, causing vertical displacements of the ocean thermocline to be larger than the lake’s for a given IGW energy (Eq. 3). In fact, open-ocean IGWs reach hundreds of meters in amplitude and can travel for thousands of kilometers^12,13,25^. To explore the potential impact of landslide-generated IGWs on oceanic energy budgets, we consider the Storegga landslide as an extreme scenario. Using previously published values of its volume (*V* = 2400 km^3^), runout distance (*R*_*a*_ = 150 km) and the mean slope of the seabed (*S* = 1%)^26^, we assume a sediment density ${\rho }_{slide}=2000\,{\rm{kg}}\,{{\rm{m}}}^{-3}$ and estimate its total potential energy as $g(V{\rho }_{slide})\cdot ({R}_{a}S)\sim 7\times {10}^{19}\,{\rm{J}}$. If 1% of this energy were transferred to IGWs and distributed evenly over the Norwegian Sea (area = 1.4 × 10^6^ km^2^), the resulting anomaly in baroclinic energy would have a mean area density of 5 × 10^5^ J m^−2^, or more than 100 times that of the mean global IGW field^27,28^. Under these assumptions and a linear two-layer formulation (Eq. 3) where $({\rho }_{2}-{\rho }_{1})=2.8\,{\rm{kg}}\,{{\rm{m}}}^{-3}$ is the modern-day difference between Norwegian sea water density at the surface and 500 m depth^29^, the Storegga landslide would have covered 9% of the Arctic Ocean with internal tsunami waves of amplitude 190 m (380 m crest to trough). While surface tsunami waves can drastically transform coastal environments, the energy of internal tsunami waves is largely confined to the ocean interior, where it may drive anomalous turbulent mixing (Fig. 1D) and thus alter large scale buoyancy-driven currents.

The coarse resolution of our bathymetry data (Supp. Fig. 1) does not allow us to make a precise calculation of the potential energy released by landslides in SMO. However, for a rough estimate of energy transfer into IGWs, we can calculate the potential energy ${E}_{slide}={M}_{slide}g{\rm{\Delta }}h$ released by the collapse of a sediment mass *M*_*slide*_ from Δ$h=20\,{\rm{m}}$ above the lake bottom. Placing an upper bound of 4.1 × 10^5^ m^3^ on the landslide volume (5% the total volume of Agua Caliente Bay, Fig. 2B) and assuming a maximum slide density ${\rho }_{slide}=2000\,{\rm{kg}}\,{{\rm{m}}}^{-3}$ yields ${E}_{slide}=1.6\times {10}^{11}\,{\rm{J}}$. Using the maximum wave energy estimated from P2 data (Fig. 1C), this translates to a minimum rate of energy transfer into thermocline IGWs of ${E}_{IGW}/{E}_{slide}=1.1\times {10}^{9}/1.6\times {10}^{11}=0.7 \%$. While this lower bound calculation is necessarily imprecise, it demonstrates the plausibility of our estimates for the Storegga landslide. Overall, this underscores how extreme geophysical events could potentially disrupt regional patterns in oceanic heat transport and turbulent mixing via the generation of internal tsunami waves. For now, the large-scale impacts of subsurface ocean motions triggered by submarine landslides remain uncertain but are likely to motivate future research.

## Conclusions

Our analyses cannot determine the extent to which observed geomorphic transformations (Fig. 2) were caused by the events discussed above. However, even our most conservative estimates of sediment concentration at the time of onset (55 ± 15 kg m^−3^ assuming a homogeneous distribution of matter throughout the water column, Fig. 3A) are well above most measurements made in oceanic turbidity currents^30^. Moreover, frequency spectra of temperature measured after the landslide reveal the merging of two IGW modes onto a single wave of intermediate length (Fig. 5). This implies that topographic changes caused by the landslide of August 18 were large enough to qualitatively modify the lake’s intrinsic dynamical modes. Hence, Fig. 5 demonstrates that underwater landslides can be diagnosed using moored observations of IGWs not only through the episodic generation of internal tsunami waves, but also via abrupt, long-lasting changes in their propagation characteristics. Alternate methods for sampling submarine mass movements are highly desirable, as some giant submarine landslides do not generate large surface waves and thus may not be identified by tsunami warning systems^6,31^.

We conclude that our observations detail the hydrodynamic response to underwater landslides that reshaped volcanic lake Santa María del Oro over the summer of 2006. These events triggered large-amplitude baroclinic waves that propagated along vertical gradients in temperature (thermocline IGWs) and suspended matter (turbid internal seiches) while transporting mass and energy away from their generation site. Our direct measurements show that, in the presence of a background density stratification, thermocline IGWs can lift and redistribute sediment at shallow depths but are also coupled to the propagation of basal turbidity currents that hold the bulk of sediment released by landslides. Able to propagate beyond topographic boundaries that confine turbidity currents, the shallow transport of sediment by thermocline IGWs may expand the influence area of sedimentary processes tied to submarine landslides and has yet to be represented in numerical models. An order of magnitude calculation indicates that thermocline IGWs received roughly 0.7% of the potential energy released by the largest landslide in our record, leading to enhanced vertical mixing characterized by a 17% decrease in the lake’s density stratification and a basin-averaged turbulent dissipation rate of $\varepsilon =3\pm 0.3\times {10}^{-8}\,{\rm{J}}\,{{\rm{kg}}}^{-1}$. These calculations suggest that baroclinic motions triggered by large submarine landslides could plausibly impact the large scale, buoyancy-driven oceanic currents that shape climate on Earth.

While the mechanisms that triggered the landslides observed in this experiment remain unknown, the temporal proximity (less than 2 months) between both major events suggests they resulted from a gradual process of slope erosion and destabilization in Agua Caliente Bay. Furthermore, local subsidence at P1 stopped hours before the landslide of June 25, until a second sediment cloud passed through this site and generated a large, short-lived disturbance in bottom pressure around 4 AM of June 27 (Fig. 4A). This indicates that the slow deepening of the lakebed observed over the first days of our record (Fig. 1B) may have been mechanically related to larger failures in this data set. Although groundwater is thought to flow into SMO, the lake’s water chemistry^32^ suggests that significant gas release that could render the slope unstable is unlikely. Instead, we believe that seasonal runoff entering the lake from surrounding hills may have contributed to destabilize the Agua Caliente Bay slope. Alternatively, observations and theory have shown that near-bottom baroclinic flows can drive slope erosion and ultimately shape the margins of stratified basins^16,33^. Given the persistence of a wind-driven, baroclinic circulation in SMO, the effects of internal wave loading on slope erosion and destabilization cannot be ruled out as contributors to sediment failure.

## Methods

Subsurface measurements of pressure locate our instruments relative to the lake surface. To remove the effects of a changing water level, we subtracted low-pass filtered fluctuations of pressure at P2 from our raw time series of bottom pressure at P1. The resulting record is shown in Fig. 1C; its temporal variability results from both hydrostatic and non-hydrostatic processes including IGWs, sediment resuspension and sensor displacement. We consider the contribution of surface waves to be negligible, as the averaging period of our instrument (6 min) exceeds that of surface seiches (2.6 min)^18^. Temperature data collected by the thermistor array in Fig. 1A further allow to infer contributions made by the lake’s time-dependent density structure. Using data from the P2 chain, we calculated water temperature at all depths by extrapolating measurements to mean bottom and surface temperatures obtained from 19 full-depth casts taken on August 20 and 21 of 2006. Next, the resulting temperature profiles were used with ADCP observations of vertical velocity to calculate the hydrostatic *p*_*h*_ and non-hydrostatic *p*_*nh*_ pressure effects of the lake’s IGW field^34,35^.

When estimated bottom pressure anomalies $({p}_{h}+{p}_{nh})^{\prime}$ are one order of magnitude smaller than the signal ${p}_{sed}={p}_{m}-{p}_{h}-{p}_{nh}$, we can isolate the effects of suspended sediment on our direct measurements *p*_*m*_. Considering the water column above P1 as a static mixture of water and sediment with densities ${\rho }_{water}$, ${\rho }_{sed}$ and vertically-integrated concentrations ${\rho }_{water}(H-h)$, ${\rho }_{sed}h$ so that *H* is the height of the water column, we can write bottom pressure as

1 $$\begin{array}{rcl}{p}_{m} & = & g{\rho }_{water}(H-h)+g{\rho }_{sed}h\\  & = & g{\rho }_{water}H+({p}_{h}+{p}_{nh})^{\prime} +{p}_{sed}.\end{array}$$

Thus, when we ignore the contributions of $({p}_{h}+{p}_{nh})^{\prime}$, the vertically-integrated concentration of sediment above P1 is calculated as

2 $${\rho }_{sed}h={\rho }_{sed}\frac{{p}_{sed}}{({\rho }_{sed}-{\rho }_{water})g}.$$

The linear estimate of thermocline energy in Fig. 1B uses the two layer formulation in equation (3), where mean densities ${\rho }_{1}$, ${\rho }_{2}$ of the upper and lower layers were obtained from the extrapolated P2 data. Thermocline displacements $\zeta$ were calculated using equation (4), which compares the background thermal stratification $\overline{\frac{\partial T}{\partial z}}$ to temperature fluctuations $\frac{\partial T}{\partial t}$ recorded by sensors at 14 m depth inside Agua Caliente Bay (the red, dashed rectangle in Fig. 1A). The nonlinear estimate of APE^36,37^ was derived using the full-column density profiles extrapolated from P2 data, thus capturing energy associated with strain and nonlinearities in the lake’s true density stratification. Both estimates of APE were low-pass filtered with a 4.1 h cutoff to remove phase effects and better represent the mean energy of thermocline oscillations throughout the basin. After comparing estimates of APE to ADCP measurements of kinetic energy at P1, we made the assumption that 85% of internal energy in this area is in the form of potential energy, so that $E=AP{E}_{ACB}/0.85\,\ast \,thermocline\,area$ gave us the basin-integrated energy of thermocline oscillations (Fig. 1C). Estimates of the squared buoyancy frequency ${N}^{2}=-\,\frac{g}{\rho }\frac{\partial \rho }{\partial z}$ used in Fig. 5 were derived from the density stratification at T1, which was multiplied by 1.6 to reproduce the average stratification as resolved by the P2 chain (Fig. 1D)^38^.

3 $$APE=({\rho }_{2}-{\rho }_{1})g{\zeta }^{2}/2$$

4 $$\frac{\partial \zeta }{\partial t}={(\overline{\frac{\partial T}{\partial z}})}^{-1}\frac{\partial T}{\partial t}$$

## Supplementary information

Supplementary material

## References

[1] Synolakis CE, Bernard EN (2006). Tsunami science before and beyond boxing day 2004. Philosophical Transactions of the Royal Society of London A: Mathematical, Physical and Engineering Sciences.

[2] Tappin DR, Watts P, McMurtry G, Lafoy Y, Matsumoto T (2001). The sissano, papua new guinea tsunami of july 1998—offshore evidence on the source mechanism. Marine Geology.

[3] Synolakis, C. E. *et al*. The slump origin of the 1998 papua new guinea tsunami. In *Proceedings of the royal society of london A: Mathematical*, *physical and engineering sciences*, vol. 458, 763–789 (The Royal Society, 2002).

[4] Tappin DR (2014). Did a submarine landslide contribute to the 2011 tohoku tsunami?. Marine Geology.

[5] Sassa S, Takagawa T (2019). Liquefied gravity flow-induced tsunami: first evidence and comparison from the 2018 indonesia sulawesi earthquake and tsunami disasters. Landslides.

[6] Harbitz CB, Løvholt F, Bungum H (2014). Submarine landslide tsunamis: how extreme and how likely?. Natural Hazards.

[7] Harbitz, C. B., Løvholt, F., Pedersen, G. & Masson, D. G. Mechanisms of tsunami generation by submarine landslides: a short review. *Norwegian Journal of Geology/Norsk Geologisk Forening* **86** (2006).

[8] Toorman, E., Bruens, A., Kranenburg, C. & Winterwerp, J. Interaction of suspended cohesive sediment and turbulence. In *Proceedings in Marine Science*, vol. 5, 7–23 (Elsevier, 2002).

[9] Luchi R, Balachandar S, Seminara G, Parker G (2018). Turbidity currents with equilibrium basal driving layers: A mechanism for long runout. Geophysical Research Letters.

[10] Rimoldi B, Alexander J, Morris S (1996). Experimental turbidity currents entering density-stratified water: analogues for turbidites in mediterranean hypersaline basins. Sedimentology.

[11] Hammack JL (1980). Baroclinic tsunami generation. Journal of Physical Oceanography.

[12] Alford MH (2003). Redistribution of energy available for ocean mixing by long-range propagation of internal waves. Nature.

[13] Zhao Z, Alford MH, Girton JB, Rainville L, Simmons HL (2016). Global observations of open-ocean mode-1 m2 internal tides. Journal of Physical Oceanography.

[14] Pomar L, Morsilli M, Hallock P, Bádenas B (2012). Internal waves, an under-explored source of turbulence events in the sedimentary record. Earth-Science Reviews.

[15] MacKinnon JA (2017). Climate process team on internal wave–driven ocean mixing. Bulletin of the American Meteorological Society.

[16] Cacchione D, Pratson LF, Ogston A (2002). The shaping of continental slopes by internal tides. Science.

[17] Sawyer DE, Mason RA, Cook AE, Portnov A (2019). Submarine landslides induce massive waves in subsea brine pools. Scientific reports.

[18] Serrano, D., Filonov, A. & Tereshchenko, I. Dynamic response to valley breeze circulation in santa maria del oro, a volcanic lake in mexico. *Geophysical Research Letters* **29** (2002).

[19] Pantin H, Leeder M (1987). Reverse flow in turbidity currents: the role of internal solitons. Sedimentology.

[20] Waterhouse AF (2014). Global patterns of diapycnal mixing from measurements of the turbulent dissipation rate. Journal of Physical Oceanography.

[21] Whalen, C., Talley, L. & MacKinnon, J. Spatial and temporal variability of global ocean mixing inferred from argo profiles. *Geophysical Research Letters* **39** (2012).

[22] Rimoldi B (1993). Turbiditic sediments in anoxic environments of the eastern mediterranean; core kc06b (libeccio basin). preliminary results. Rendiconti Lincei.

[23] DePalma RA (2019). A seismically induced onshore surge deposit at the kpg boundary, north dakota. Proceedings of the National Academy of Sciences.

[24] Damuth JE (1979). Migrating sediment waves created by turbidity currents in the northern south china basin. Geology.

[25] Alford MH (2015). The formation and fate of internal waves in the south china sea. Nature.

[26] Bondevik, S. *et al*. The storegga slide tsunami—comparing field observations with numerical simulations. In *Ormen Lange–an Integrated Study for Safe Field Development in the Storegga Submarine Area*, 195–208 (Elsevier, 2005).

[27] Ferrari R, Wunsch C (2010). The distribution of eddy kinetic and potential energies in the global ocean. Tellus A: Dynamic Meteorology and Oceanography.

[28] Eakins, B. & Sharman, G. Volumes of the world’s oceans from etopo1. *NOAA National Geophysical Data Center*, *Boulder*, *CO* **7** (2010).

[29] Roemmich D, Gilson J (2009). The 2004–2008 mean and annual cycle of temperature, salinity, and steric height in the global ocean from the argo program. Progress in oceanography.

[30] Xu J, Sequeiros OE, Noble MA (2014). Sediment concentrations, flow conditions, and downstream evolution of two turbidity currents, monterey canyon, usa. Deep Sea Research Part I: Oceanographic Research Papers.

[31] Løvholt F, Bondevik S, Laberg JS, Kim J, Boylan N (2017). Some giant submarine landslides do not produce large tsunamis. Geophysical Research Letters.

[32] Armienta MA (2008). Water chemistry of lakes related to active and inactive mexican volcanoes. Journal of Volcanology and Geothermal Research.

[33] Canals M (2006). Flushing submarine canyons. Nature.

[34] Moum J, Smyth W (2006). The pressure disturbance of a nonlinear internal wave train. Journal of Fluid Mechanics.

[35] Moum J, Nash J (2008). Seafloor pressure measurements of nonlinear internal waves. Journal of Physical Oceanography.

[36] Holliday D, McIntyre M (1981). On potential energy density in an incompressible, stratified fluid. Journal of Fluid Mechanics.

[37] Kang D, Fringer O (2010). On the calculation of available potential energy in internal wave fields. Journal of Physical Oceanography.

[38] Brizuela, N. Acoplamiento entre ondas internas y deslizamientos en el fondo de un lago volcánico (2017).

