# Supplementary material for: Internal tsunami waves transport sediment released by underwater landslides
Source: Sci Rep. 2019 Jul 24;9:10775. doi: 10.1038/s41598-019-47080-0 (PMC6656747; doi:10.1038/s41598-019-47080-0)
Supplement: Supplementary file 1 — Supplementary material [file 41598_2019_47080_MOESM1_ESM.docx]

**Supplementary Material for ‘Internal tsunami waves transport sediment released by underwater landslides’**

Brizuela, N.^1,2,*^, Filonov, A.^2^, Alford, M.H.^1^

^1^ Scripps Institution of Oceanography, University of California San Diego, La Jolla, 92093 USA

^2^ Department of Physics, Universidad de Guadalajara, Guadalajara, 44430 Mexico

^*^ nogutier@ucsd.edu

| 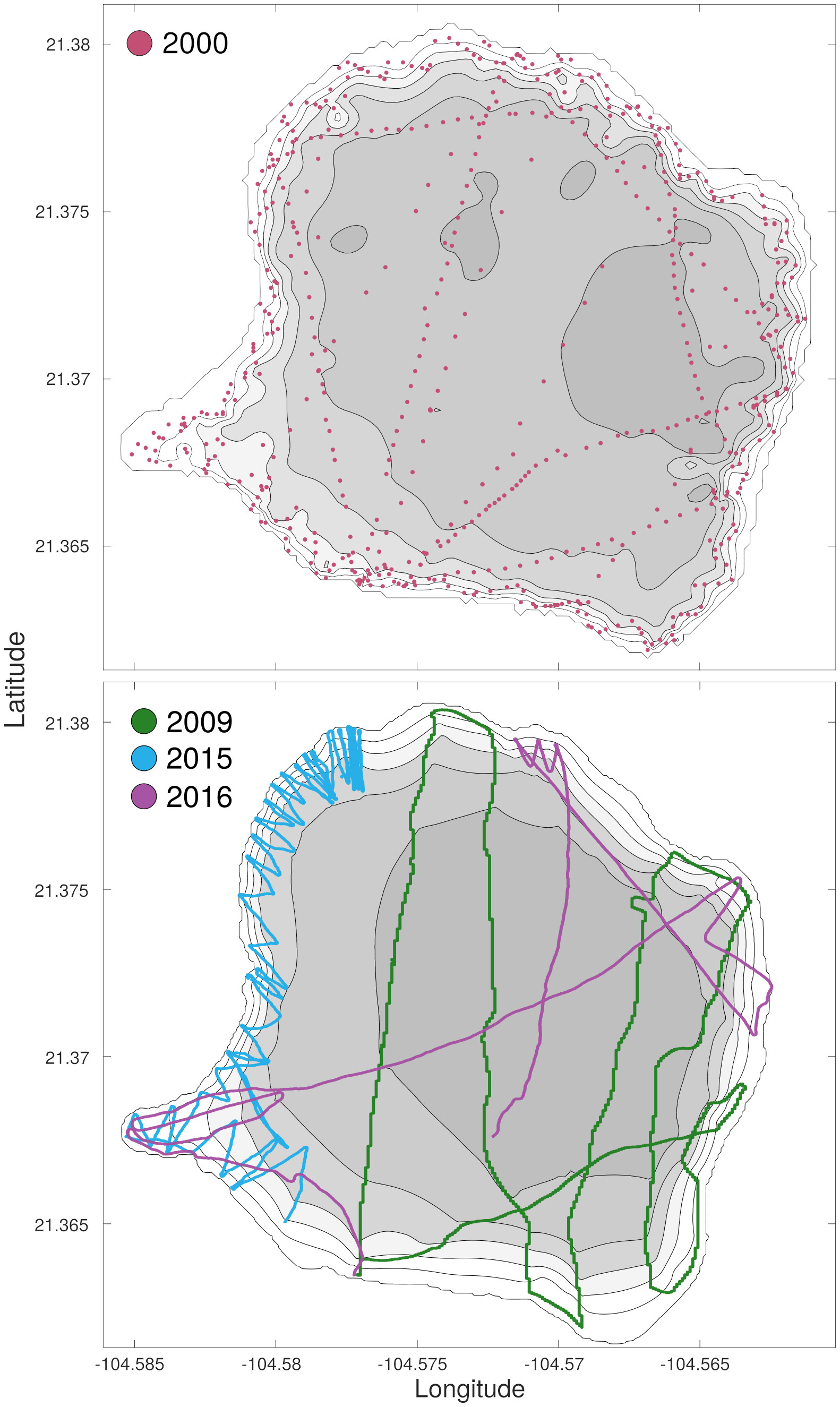 |
| --- |
| **Supplementary Figure 1: Locations of depth measurements made in 2000, 2009, 2015 and 2016.** Each set of measurements is plotted with the interpolated bathymetry map they helped produce (depth contours are shown at 10 m intervals). |
| 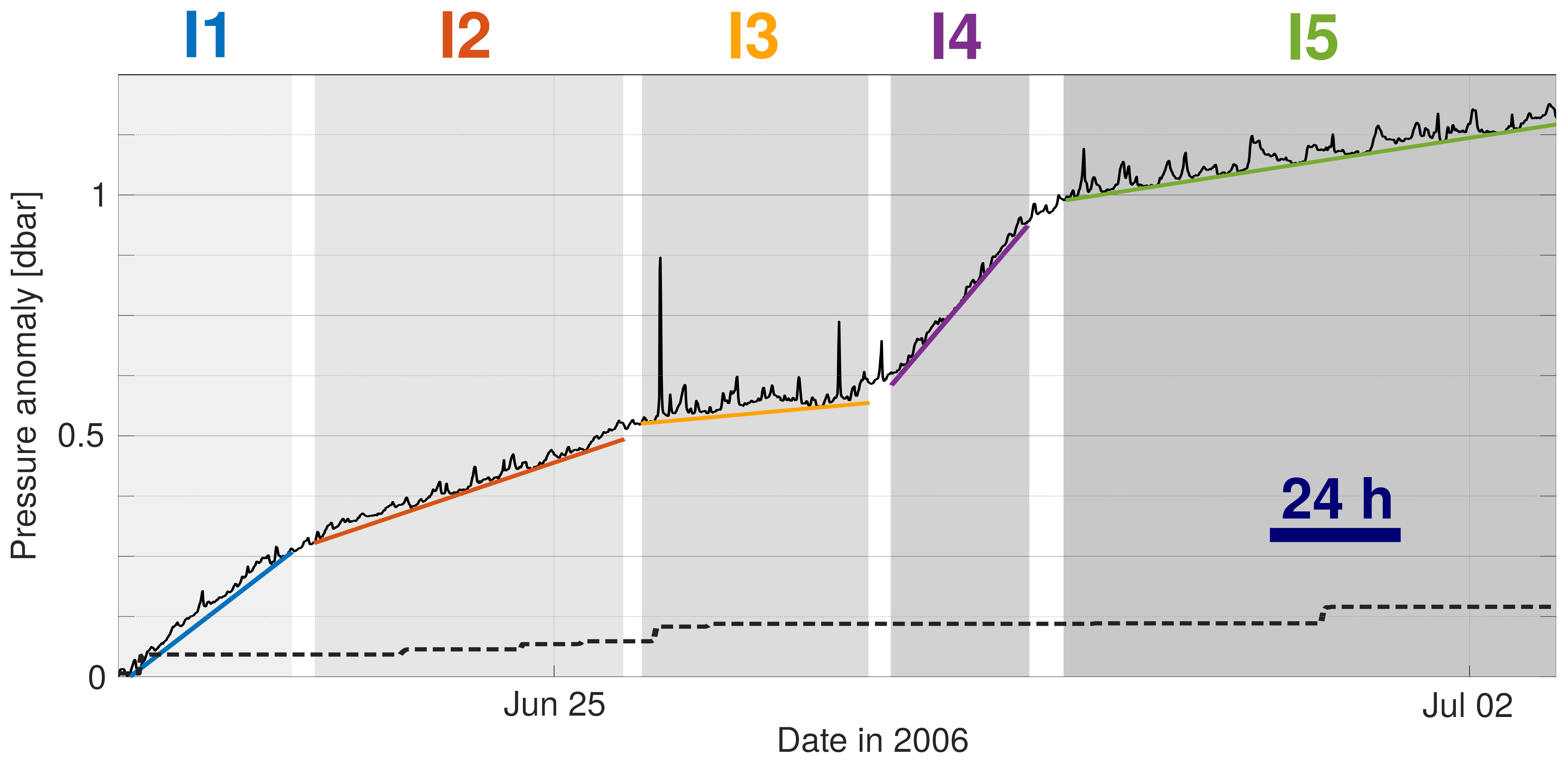 |
| **Supplementary Figure 2:** **Bottom pressure in the first 11 days of our experiment.** Changes in the raw time series of bottom pressure at P1 (black) are categorized under five distinct regimes of sediment motion. Gray shading defines time intervals (I1, I2, I3, I4, I5) for each regime. The slopes of colored lines show the mean rate of change in pressure over each interval. Contributions from rainfall to bottom pressure (dashed) are calculated using data from the weather station south of the lake (Fig. 1.A). Estimated rates of local subsidence for each interval are presented in Supplementary Table 1. |

| \| Interval \| Duration [hours] \| Change in bottom pressure [dbar] \| Contribution from rainfall [dbar] \| Downward sediment velocity [m day^-1^ ] \| \| --- \| --- \| --- \| --- \| --- \| \| **I1** \| 32 \| 0.28 \| 0.05 \| 0.17 \| \| **I2** \| 57 \| 0.22 \| 0.03 \| 0.08 \| \| **I3** \| 42 \| 0.04 \| 0.04 \| 0 \| \| **I4** \| 25 \| 0.33 \| 0 \| 0.32 \| \| **I5** \| 90 \| 0.16 \| 0.04 \| 0.03 \| |
| --- | --- | --- | --- | --- | --- | --- | --- | --- | --- | --- | --- | --- | --- | --- | --- | --- | --- | --- | --- | --- | --- | --- | --- | --- | --- | --- | --- | --- | --- | --- |
| **Supplementary Table 1: Rates of local subsidence at P1 throughout the first 11 days of our experiment.** We use linear fits in Supplementary Figure 1 to estimate the total change in bottom pressure throughout each interval. After subtracting contributions made by rainfall, we use these to estimate the daily rate of local subsidence at P1. Our calculations represent an upper bound for downward sediment velocity, as we do not account for changes in the water level induced by runoff and because our sensor may slip at a greater rate than the sediment below it. |
